# The conserved stem-loop II structure at the 3' untranslated region of Japanese encephalitis virus genome is required for the formation of subgenomic flaviviral RNA

**DOI:** 10.1371/journal.pone.0201250

**Published:** 2018-07-26

**Authors:** Yi-Shiuan Chen, Yi-Hsin Fan, Chih-Feng Tien, Andrew Yueh, Ruey-Yi Chang

**Affiliations:** 1 Department of Life Science, National Dong Hwa University, Hualien, Taiwan, ROC; 2 Institute of Biotechnology and Pharmaceutical Research, National Health Research Institutes, Miaoli, Taiwan, ROC; Universitat Bern, SWITZERLAND

## Abstract

Flaviviruses accumulate abundant subgenomic RNA (sfRNA) in infected cells. It has been reported that sfRNA results from stalling of host 5’-to-3’ exoribonuclease XRN1 at the highly structured RNA of the 3’ untranslated region (UTR). Although XRN1 digestion of a 3’-terminal 800-nt RNA could stall at a position to generate the sfRNA *in vitro*, we found that knocking out XRN1 had no effect on the accumulation of sfRNA in Japanese encephalitis virus (JEV) infected cells. Mutagenesis studies revealed that the stemloop II (SLII) at the 3’ UTR is required for the accumulation of sfRNA. According to the results of an *in vitro* RNA-dependent RNA polymerase (RdRp) assay, the (-)10431-10566 RNA fragment, containing the putative promoter on the antigenome for the sfRNA transcription, binds to RdRp protein and exhibits a strong promoter activity. Taken together, our results indicate that the JEV sfRNA could be transcribed initially and then be trimmed by XRN1 or other unidentified exoribonucleases.

## Introduction

Japanese encephalitis virus (JEV) belongs to the family *Flaviviridae* and is a mosquito-borne zoonotic pathogen that causes human encephalitis and meningitis. The 10,976 nucleotides (nts) genome contains a single open reading frame (ORF) encoding a polyprotein which is subsequently processed into three viral structural proteins and seven nonstructural proteins [1]. The ORF is flanked by 5’ and 3’ untranslated regions (UTRs) which contain many important *cis*-acting elements involved in the regulation of viral translation, replication, and pathogenesis [2–5].

During virus infection, all arthropod-flaviviruses generate abundant amounts of a noncoding subgenomic RNA (sfRNA) representing the 3’-terminal highly conserved region of the 3’ UTR [6–9]. The sfRNA engages in multiple functions in order to control viral replication and antagonize host antiviral responses [4, 10, 11]. These functions include (i) the involvement of cytopathicity and pathogenicity in mammals [8]; (ii) the down-regulation of antigenome synthesis and translation [12]; (iii) the dysregulation of host mRNA stability [13, 14]; (iv) the antagonizing of host innate immune response [15–17]; (v) the suppression of RNA silencing [18, 19]; (vi) the induction of apoptosis through the Bcl-2-mediated PI3k/Akt signaling pathway [20]; and (vii) the determination of infection and transmission rates of West Nile virus (WNV) and Dengue virus (DENV) in mosquitoes [21, 22].

How the sfRNA is made in the infected cells is intriguing. Several studies have shown that the sfRNA is a product of the incomplete degradation of the viral genome by cellular 5’-to-3’ exoribonuclease XRN1; a pseudoknot or a three-helix junction structure in the 3’ UTR is responsible for stalling XRN1 degradation [8, 23–26]. This cleavage happened within a few minutes when carried out *in vitro*, and there were no intermediate byproducts during the exoribonuclease degradation process of the viral genome to the sfRNA in the virus-infected cells, which indicated that the degradation process is extremely efficient or that another biogenesis mechanism might be involved. The reason for these findings is not clear because degradation from the full-length genome seems uneconomic for viruses from an evolutionary point of view. Furthermore, being infected with RNA viruses induces double membrane vesicles to form replication/transcription complexes that are protease- and nuclease-resistant [27–29]. The reason for such abundant levels of viral genome within the membrane-protected complex subjected to RNA degradation remains unclear. Although RdRp has no proofreading activity, considering the high molar ratio of the sfRNA to the genome (1.25–5.14 in mosquito cells and 0.25–1.5 in mammalian cells) [9], the possibility that large portions of the synthesized genome are dysfunctional and subjected to degradation pathways is elusive. It may just be that the RNA turnover by XRN1 exoribonucleases is quite normal but that sfRNA accumulates because it is degradation resistant.

In this study, we found that either knocking down or knocking out of exoribonuclease XRN1 had no effect on the accumulation of sfRNA in JEV-infected cells. In contrast, mutation of GDD motif in RdRp and disruption of the SLII structure halted accumulation of the sfRNA. Furthermore, the minus-strand templates covering the putative promoter region used for an *in vitro* RdRp assay gave rise to synthetic products, suggesting that the JEV sfRNA could be initially transcribed from the antigenome and may be further trimmed by XRN1 or other unidentified exoribonucleases.

## Materials and Methods

### Cells and virus

BHK-21 and A549 cells were grown at 37°C in RPMI 1640 medium supplemented with 2% and 10% fetal bovine serum (FBS, GIBCO), respectively. A549 XRN1 knock-out cells were kindly provided by Dr. Bernard Moss (NIAID, NIH) and were propagated as described previously [30]. Human embryonic kidney (HEK293T) cells were grown in Dulbecco’s Modified Eagle’s Medium (DMEM, GIBCO) supplemented with 10% FBS (GIBCO). JEV strain RP9 (GenBank accession number AF014161) and Dengue virus type 2 (DENV-2) strain 16681 were used in this study.

### Plasmids and site-directed mutagenesis

Detailed methods for the construction of clones are described in the Supporting Information S1 Text. The majority of plasmids used for this study were constructed by RT-PCR using synthetic oligonucleotides, as listed in S1 Table. The cDNA encoded RdRp domain from amino acids at position 274 to 905 of the JEV NS5 protein was amplified by RT-PCR and cloned into pET28a (Novagen).

### XRN1 RNA interference, Western and Northern analyses

The lentiviral vectors expressing the short hairpin RNA (shRNA) (TRCN-296739) targeting human XRN1 gene (GenBank accession no. NM_019001.3) or a negative control shRNA targeting GFP (shGFP) were obtained from the Taiwan National RNAi Core Facility. To knockdown XRN1 expression, 5 × 10^5^ HEK293T and A549 cells were transduced with the shXRN1 or shGFP lentiviruses, respectively, and selected with puromycin (1 μg/ml). At one-week post-transduction, cells were seeded on 60-mm plates (approximately 2 × 10^6^ cells per plate) and infected with JEV RP9 (or DENV-2 as a control) at an MOI of 5 (for HEK293T) or 10 (for A549). The cells were harvested with PBS at 48 h post-infection, and the precipitates were then divided into two parts. One part was analyzed for Western blotting and the other part was used for Northern analysis. Equivalent amounts of proteins (20 μg/sample) were resolved by 8% SDS-PAGE, followed by Western blotting using anti-XRN1 (1:1000, Sigma) or anti-β actin (1:5000, Sigma) antibodies. RNA extraction and Northern analyses were conducted as described previously [9, 15]. In some cases, the blots were hybridized with the IRD 700-labeled JEV(-)10950-10976 oligonucleotide, and the signals were scanned on a Typhoon 9000 imaging scanner (GE Healthcare); otherwise, the blots were hybridized with the strand-specific digoxigenin (DIG)-labeling riboprobe detecting JEV nt 10454–10976 or DENV-2 nt 10270–10732 made by *in vitro* transcription (Roche Molecular Biochemicals) and then visualized using luminescent image analyzer (LAS-3000, Fujifilm), as described in a previous study [12]. The relative intensities of RNA bands on the membrane were estimated using Multi Gauge software (Fujifilm).

### Nuclease assays

Plasmid pGEMT-JEV-800 was linearized with *Sal* I and transcribed *in vitro* using T7 RNA polymerase (Promega) according to the manufacturer’s protocol. DNA template used for run-off transcription to generate 3’-terminal 800 nts of DENV was amplified from pTight-DENV plasmid [31] using oligonucleotides F10 and R10 as shown in Supplemental S1 Table. DNA template was removed by DNase I. Approximately 60 pmol of triphosphated RNAs were incubated with 20 units of RNA 5’ pyrophosphohydrolase (RppH, NEB) at 37°C for 2 hours to generate 5’-monophosphated RNA. The RppH-treated RNA was purified by phenol-chloroform extraction and ethanol precipitation. Next, 500 ng of the monophosphated RNA was digested with the indicated amount of XRN1 (NEB) or RNase A (Qiagen) for 1 hour at 37°C. 200 ng of RNA was then separated in a formaldehyde-containing 1.5% agarose gel and hybridized to a riboprobe as indicated.

### Transfection

The procedure for DNA-based infectious clone transfection has been described previously [31]. Briefly, BHK-21 cells (3 × 10^4^ cells/well) in 12-well plates were transfected with 0.25 μg of pTight-JEV (or mutant) plasmid, 0.25 μg of pTET-OFF (BD Bioscience), and 1 μl of Lipofectamine 2000 (Invitrogen) in 200 μl of Opti-MEM medium per well at 37°C for 4 hours, and the cells were refed with fresh medium. Total RNAs were extracted at 5 days post-transfection (dpt), and the supernatant fluids were collected for measuring the virus titers. The transfected cells were passaged every 5 days if no apparent cytopathic effect (CPE) was observed. RNAs were extracted at the time when the cells showed a severe CPE.

### Plaque assay

The titration of infectious virus was performed as previously described [32]. Briefly, serial 10-fold dilutions of virus were made in serum-free alpha-MEM medium, and 0.1 ml of each dilution was added per well to a monolayer of BHK-21 cells in a 12-well plate. After 1 hour of absorption at 37°C, the cells were washed with PBS and overlaid with growth medium containing 0.3% LE agarose (Lonza), and the plaque was visualized with naphthol blue–black solution (0.1% naphthol blue–black, 1.36% sodium acetate and 6% glacial acetic acid) at 4 days post-infection. Viral titers were determined as plaque forming units per milliliter.

### Electrophoretic mobility shift assay (EMSA)

Riboprobe was made by *in vitro* transcription with T7 RNA polymerase (Promega) and DIG-UTPs (Roche Molecular Biochemicals). The DIG-labeled RNAs (0.25 pmol) were denatured at 80°C for 10 min and then quick cooling on ice. Binding reactions were set at room temperature in binding buffer (10 mM HEPES pH 7.1, 20 mM KCl, 1 mM MgCl_2_, 1 mM DTT, 10 units RNaseOUT) with purified recombinant RdRp protein or bovine serum albumin (BSA) at different concentrations (0.25, 0.5, 1, and 2.5 μM) in a volume of 20 μl for 30 min, followed by adding 2 μl heparin (25 mg/ml) for 10 min. The RNA-protein complexes were resolved by non-denaturing 5% polyacrylamide gel at 4°C, transferred to a positively charged nylon membrane (Amersham), and then cross-linked using UV Stratalinker (Stratagene). Detection of DIG-labeled RNA was performed using DIG Luminescent Detection Kit (Roche) according to the manufacturer’s instructions. To demonstrate the specificity of RNA-protein complex formation, competition assays were performed using various amounts of unlabeled RNAs added to the binding reaction.

### *In vitro* RdRp assay

A recombinant JEV RdRp was expressed in *E*. *coli* Rosetta (Novagen). Protein expression was induced with isopropyl-β-D-thiogalactopyranoside (IPTG) at a final concentration of 0.5 mM and induced culture was grown at 22°C for 15 hours. Expressed RdRp was purified by metal affinity chromatography on a nickel-chelate column according to the manufacturer’s instructions (Invitrogen). The RNA templates used for the RdRp assay and the RNA size markers were synthesized by *in vitro* transcription (Promega) in the absence or presence of α-^32^P-CTP, respectively. The *in vitro* RdRp assay was performed with 500 ng of the recombinant RdRp protein and 200 ng of RNA templates or as indicated in a total volume of 25 μl of the reaction buffer containing 35 mM Tris-HCl (pH 7.9), 50 mM NaCl, 25 mM potassium glutamate, 5 mM MgCl_2_, 1 mM DTT, 5 mM MnCl_2_, 10% glycerol, 20 units of RNase inhibitor (Promega), 5 mM (each) ATP, UTP, and GTP, 0.5 mM CTP, and 10 mCi of α-^32^P-CTP (3,000 Ci/mmole; MP Biomedicals). The reaction mixture was incubated at 30°C for 2 hours. RdRp reaction product was denatured in 2X loading buffer (95% formamide, 0.025% xylene cyanol, 0.025% bromophenol blue, 18 mM EDTA, 0.025% SDS) and resolved on 6% polyacrylamide gel containing 6M urea. The gel was dried and visualized by autoradiography.

## Results

### Abundant amounts of the JEV sfRNA accumulated in XRN1-depletion cells

**Fig 1:**
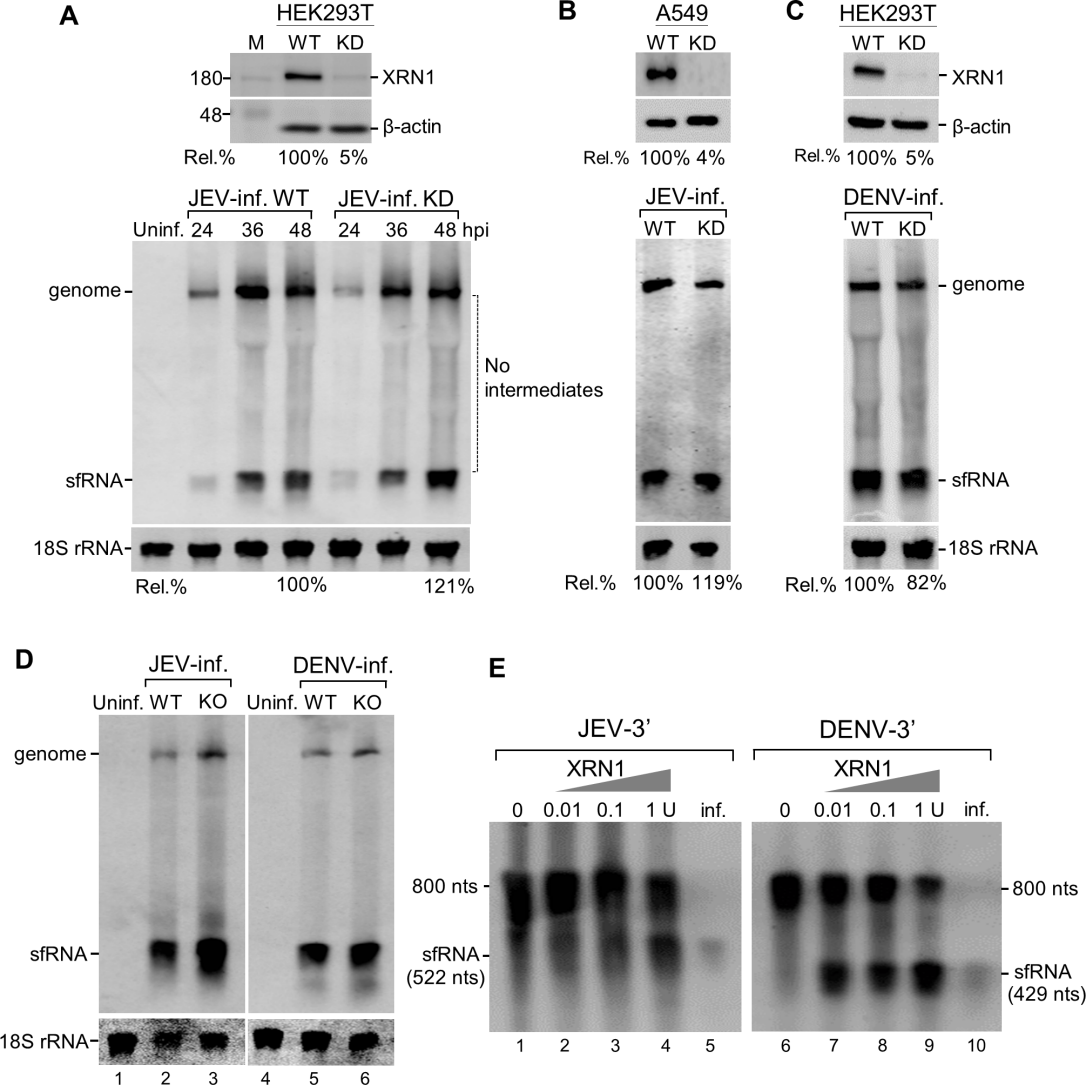
Both knocking-down and knocking-out of XRN1 had no effect on the accumulation of sfRNA. XRN1-knockdown (KD) cells were prepared as described in Materials and Methods. Knockdown efficiency was determined by Western blot using anti-XRN1 and anti-β-actin antibodies. The Rel. % value represents the percentage of XRN1 expression in cells transfected with shXRN1 compared to wild-type cells (WT)(100%) as shown at the top (A-C). The XRN1-KD HEK293T (A) or A549 (B) cells were infected with JEV. Total RNA were extracted at the indicated times post infection and Northern blots were done with a DIG-labeled riboprobe detecting nt 10454 to nt 10976 in the 3’UTR (A, D, E) or an IRD 700-labeled JEV(-)10950-10976 probe (B). (C) XRN1-KD HEK293T cells were infected with DENV-2 at an MOI of 5 as a control. Total RNAs were extracted at 72 h post-infection and Northern blots were analyzed using a DIG-labeled riboprobe detecting nt 10270 to nt 10723 in the 3’UTR. Relative amounts of sfRNA were quantified (%) in the XRN1-depleted cells. (D) XRN1-knockout (KO) cells were infected with JEV or DENN-2 at an MOI of 5. RNA isolated from these cells at 48 h post-infection was subjected to Northern blot analysis. (E) RNA degradation analysis of non-replicative 800-nt 3’-terminal monophosphate transcripts derived from genome of JEV or DENV as indicated was measured *in vitro* by incubating with the indicated amounts of XRN1. Total RNAs extracted from JEV or DENV-2 infected cells (1 μg) were used as the sfRNA size marker (lanes 5 and 10). RNAs were separated by denaturing gel and analyzed by Northern hybridization.

To investigate whether the JEV sfRNA is an exoribonuclease XRN1 cleavage product, we analyzed the effect of XRN1 depletion caused by RNA interference on sfRNA generation in virus-infected cells. Knocking-down (KD) the XRN1 expression by up to 95% in HEK293T cells continued to increase the abundance of sfRNA throughout the 48-h infection period. More sfRNA (121%) was observed in XRN1 knocked-down cells than in the wild-type (WT) cells (Fig 1A). It should be noted that there were no decay intermediates between viral genome and the sfRNA even in the XRN1-deficient cells. Similar results were also observed in A549 cells infected with JEV (Fig 1B), whereas DENV infection caused a slight reduction of sfRNA accumulation in the XRN1-KD cells (Fig 1C). Statistical analysis of JEV sfRNA accumulation in XRN1-KD cells among three independent experiments showed about 112.5% ± 8.5% at 48 h post infection. No statistically significant differences in the amount of the sfRNA were detected in the infected cells at any time in relation to the depletion of XRN1. These results indicated that either the exoribonuclease XRN1 in JEV-infected cells could be extremely active, such that the residual XRN1 (4–5%) might be sufficient to digest the JEV genome or that another nuclease or mechanism might be involved in the formation of sfRNA. To rule out the possibility that the residual XRN1 may contribute to the formation of sfRNA, we performed the experiments using XRN1 knock-out (KO) cells. As shown in Fig 1D, sfRNA accumulated abundantly in XRN1-KO cells infected with either JEV or DENV indicating that XRN1 does not play a direct role for the formation of sfRNA *in vivo*.

To determine whether purified exoribonuclese XRN1 is able to stall at a position to generate the sfRNA *in vitro*, a 3’-terminal 800-nt monophosphate RNA derived from genome of JEV or DENV was incubated with 0.01, 0.1, or 1 unit of XRN1, respectively, and analyzed by Northern blot analysis for sfRNA production. The DENV sfRNA is readily formed when treated with XRN1 at concentration as low as 0.01 units (Fig 1E, lane 7), whereas the 800-nt JEV RNA was relatively resistant to low concentrations of XRN1 (0.01 and 0.1 unit) (Fig 1E, lanes 2 and 3). In contrast, incubation with 1 unit of exoribonuclease XRN1 resulted in the production of the sfRNA (Fig 1E, lane 4), indicating that a higher concentration of exoribonuclease XRN1 is required for stalling at a position to generate JEV sfRNA. The 800-nt RNA treated with RNase A was rapidly and completely degraded (data not shown).

### The stem-loop II (SLII) structure is required for the formation of the sfRNA

**Fig 2:**
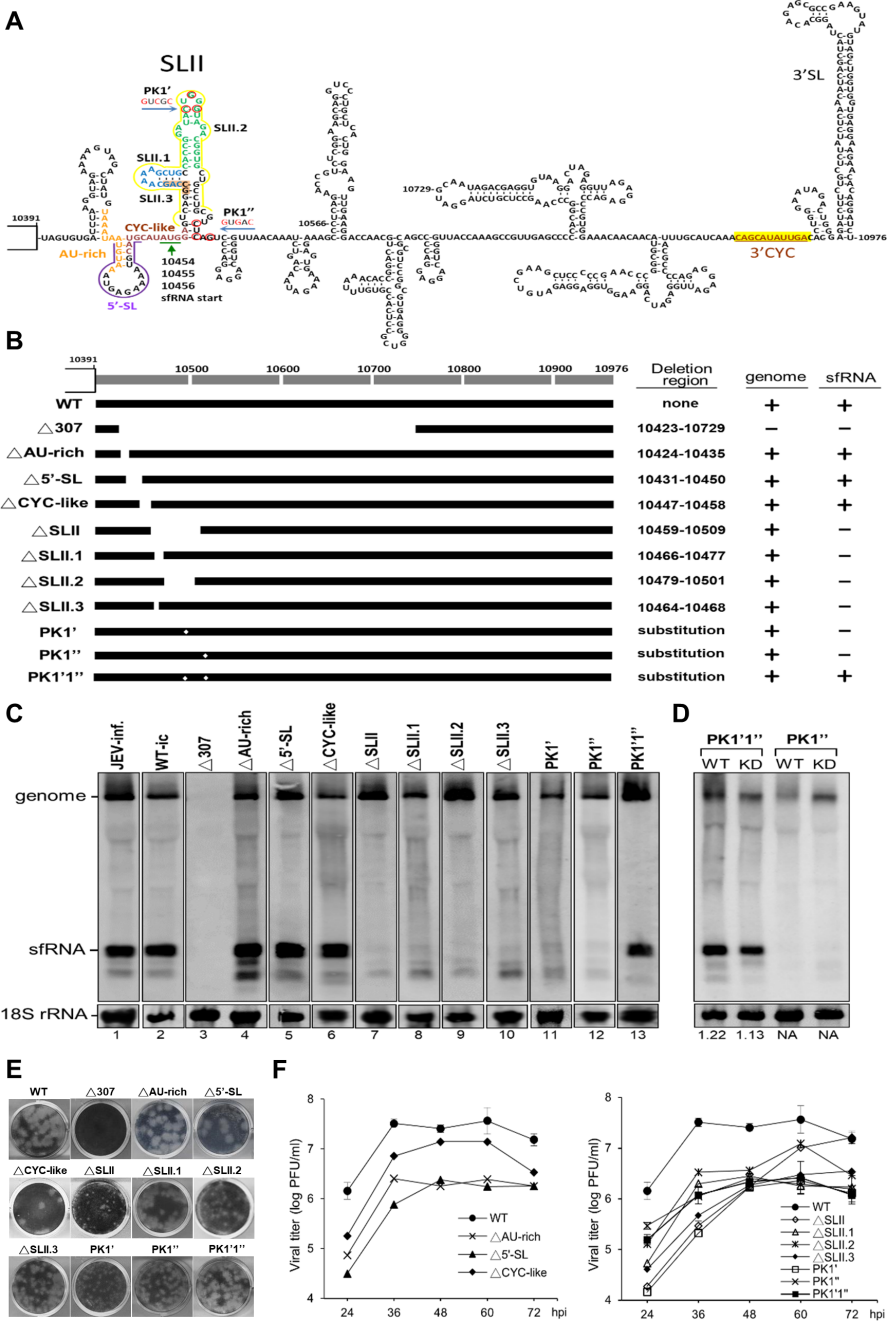
Determine the region of 3’ UTR involved in the accumulation of sfRNA constructed in the context of a full-length infectious JEV clone. (A) Predicted secondary structures within the 585-nt 3’ UTR of JEV genome. Nucleotides are numbered from the first base of the genome. The deletion or base substitution regions made in this study are marked or color-coded. (B) Schematic diagram of the 3’ UTR mutants and the results of Northern hybridization for detecting the genome and sfRNA are summarized on the right. (C) BHK-21 cells were infected with recombinant mutant viruses at an MOI of 0.1. RNAs were extracted at 48 h post-infection and Northern hybridization was done as described in Fig 1A. (D) HEK293T cells (WT) or XRN1-knockdown cells (KD) were infected with a PK disrupted mutant (PK1”), and a PK compensatory changed mutant (PK1’1”) viruses at an MOI of 1. Cytoplasmic RNAs were extracted at 60 h post-infection and analyzed by Northern blot. (E) Plaque assays of WT and all mutant viruses were done on BHK-21 cells. Cells were fixed and stained with naphthol blue–black dye at 4 days post-infection. (F) Viral growth kinetics of the WT infectious clone and the recombinant mutant viruses. BHK-21 cells were infected at an MOI of 0.1, and the supernatant fluid of the infected cells was sampled at the indicated times post infection. Titers were determined by plaque assays on BHK-21 cells.

Several studies suggest the existence of XRN1-resistant structure (xrRNA) for the production of sfRNA. To investigate the structure requirements for the production of JEV sfRNA *in vivo*, we performed mutagenesis studies in the context of a JEV infectious clone (in the S1 Text and S1 Table). The structure of the JEV 3’ UTR is diagramed in Fig 2A. Although equal amounts of cDNA (250 ng) for all mutants were transfected into BHK-21 cells, according to immunofluorescence assay (IFA) and Northern hybridization results at 5 days post-transfection (dpt), the replication efficiency of the cDNA from different mutants varied (data not shown). These results suggest that the mutations made in these regions delayed the completion of the viral life cycle. We therefore collected the supernatant and measured the virus titers at the time when the cells showed strong CPE that varied from 4 to 11 dpt for different mutants (Table 1). We then used equal amounts of MOI at 0.1 for each recombinant virus to infect BHK-21 cells and analyzed the RNA synthesis at 48 h post-infection (hpi). A deletion that spanned nt 10423–10729 (Δ307) abolished the viral replication (Fig 2C, lane 3), while the deletion of the AU-rich region (ΔAU-rich) or the 5’-stemloop (Δ5’-SL) slightly delayed viral replication but had no lasting effect on the viral replication overall or on the sfRNA accumulation (Fig 2C, lanes 4–5 and Table 1).

**Table 1:** Virus titer^a^ in supernatant fluids collected at 5 days post-transfection (dpt) and at the time of post-transfection showed a strong CPE for each cDNA construct.

Construct	Titer (pfu/ml) measured at 5 dpt^b^	Sampling time when showed strong CPE	Titer (pfu/ml) measured at the time showed strong CPE^c^
WT	8.50 × 10^5^	4 dpt	1.35 × 10^6^
Δ307	0	11 dpt	0
ΔAU-rich	0	9 dpt	1.90 × 10^5^
Δ5’-SL	1.55 × 10^5^	6 dpt	9.75 × 10^5^
ΔCYC-like	3.05 × 10^3^	6 dpt	4.50 × 10^5^
ΔSLII	0	10 dpt	5.58 x 10^5^
ΔSLII.1	0	8 dpt	3.65 × 10^5^
ΔSLII.2	0	8 dpt	4.40 × 10^5^
ΔSLII.3	3.25 × 10^5^	6 dpt	5.15 × 10^5^
PK1’	0	10 dpt	2.48 × 10^4^
PK1”	2.35 × 10^5^	6 dpt	4.70 × 10^5^
PK1’1”	3 × 10^4^	6 dpt	5.25 × 10^5^

^a^Virus assays on BHK-21 cells using an agarose overlay.

^b^Cells were passaged at five days post-transfection (dpt) and supernatant fluids were collected at the time of passaging.

^c^Sampling time for measuring the titer is indicated on the left column.

Genome cyclization has been shown to play an important role in viral replication [33]. Beside the canonical 3’-cyclization (3’CYC) motif (CAGCAUAUUGA) at 10863–10873 that is complementary to 5’CS (UCAAUAUGCUG) at nt 136–146, we found another motif located at the initiation site of JEV sfRNA that is very similar to the 3’CYC motif (named CYC-like motif), the sequence of which is CAUGCAUAUGGA, where the mismatches with the 3’CYC motif are underlined). However, deletion of the CYC-like motif had no effect on the synthesis of the sfRNA and the genome (Fig 2C, lane 6), whereas deletion of the entire SLII abolished the production of sfRNA (Fig 2C, lane 7). Meanwhile, deletion of the lower left stemloop of the SLII (ΔSLII.1), the upper stemloop (ΔSLII.2) or the lower left stem (ΔSLII.3) all impaired sfRNA synthesis (Fig 2C, lanes 8–10), indicating that the SLII structure is essential for sfRNA accumulation. Three point mutations disrupted base pairing of the first pseudoknot at the 3’ UTR (PK1’ or PK1”) prevented sfRNA formation (Fig 2C, lanes 11–12), while compensatory changes restoring the PK pairing (PK1’1”) recovered the sfRNA synthesis (Fig 2C, lane 13) suggesting that pseudoknot structure is also required for the sfRNA formation. To further demonstrate that the production of sfRNA requires the PK structure but does not rely on the activity of exoribonuclease XRN1, PK1’1” and PK1” mutants were used to infect WT or XRN1-KD HEK293T cells, and then the resulting effects were compared. Disruption of the PK structure (PK1”) abolished the sfRNA production in WT as well as in XRN1-KD cells, while compensatory changes that maintained the PK structure (PK1’1”) made no difference in the production of sfRNA in XRN1-KD cells or in WT cells (Fig 2D), indicating that the requirement of the PK structure for the production of sfRNA does not rely on the activity of exoribonuclease XRN1.

All mutations made at the SLII region apparently resulted in relatively smaller plaques (Fig 2E), suggesting that the structure or sequences of the SLII might play a role in determining the plaque size. Viral titers measured at 12-h intervals showed about 2- to 120-fold reductions in comparison with the WT control for all the mutants except for the SLII deletion mutant, which exhibited slow growth at earlier time points but eventually reached to the WT-virus growth level at 72 hpi (Fig 2F). Mutations resulted in the reduction of virus titers, suggesting that these mutations delayed virus maturation and release rather than affecting viral replication because the RNA levels of mutants measured at 48 hpi by Northern hybridization (Fig 2C) did not show apparent differences in comparison with the WT control.

### Viral replication is essential for abundant accumulation of the JEV sfRNA

**Fig 3:**
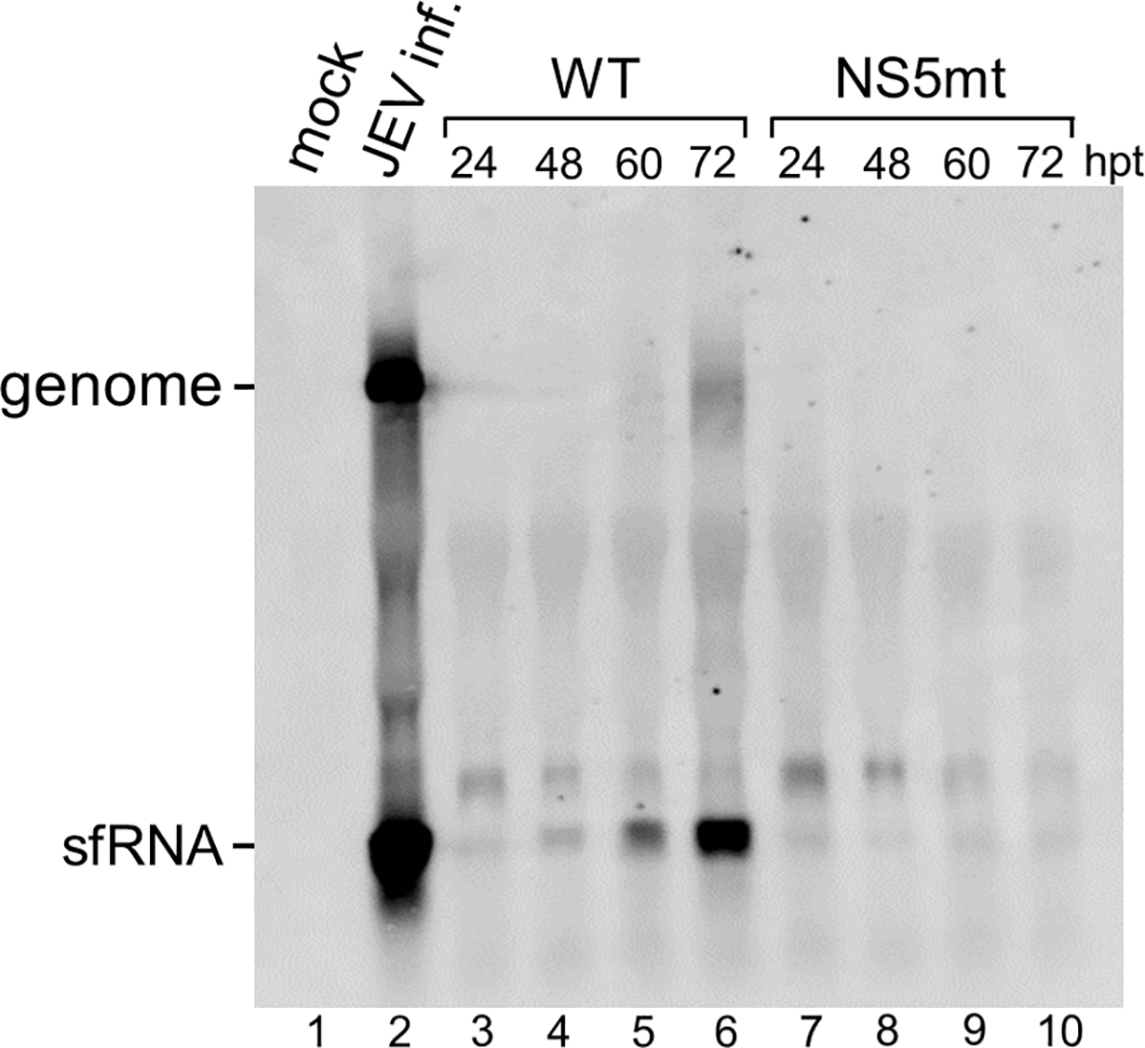
Replication of the viral genome is essential for abundant accumulation of the sfRNA. BHK-21 cells were mock-infected (lane 1) or infected with JEV at an MOI of 0.01, and the RNA was extracted at 36 h post-infection (lane 2). Total RNAs were isolated from BHK-21 cells transfected with a WT infectious clone or the NS5mt at the indicated hours post-transfection (hpt). RNAs were measured by Northern hybridization with a DIG-labeled riboprobe detecting nt 10454 to nt 10976 in the 3’ UTR.

To test whether the formation of JEV sfRNA requires viral replication, the replication-deficient mutant, which had the essential polymerase motif GDD mutated (NS5mt), was compared to the wild-type (WT) infectious clone. The sfRNA in WT DNA-transfected cells were visible at 24 hpt and continued to increase to a very high level at 72 hpt (Fig 3, lanes 3–6). The NS5mt clone, on the other hand, completely abolished the viral genome replication while barely any sfRNA accumulated, indicating that the formation of the JEV sfRNA correlates with viral replication (Fig 3, lanes 7–10). Although efficient RNA replication is required for the detection of any flaviviral RNAs despite which mechanism used for the sfRNA formation, our results were clearly different from the observations from WNV that BHK-21 cells transfected with replicon constructs containing various deletions had no effect on the accumulation of sfRNA when compared to the WT [8].

### Viral RdRp binds to the putative promoter and synthesizes RNA products

**Fig 4:**
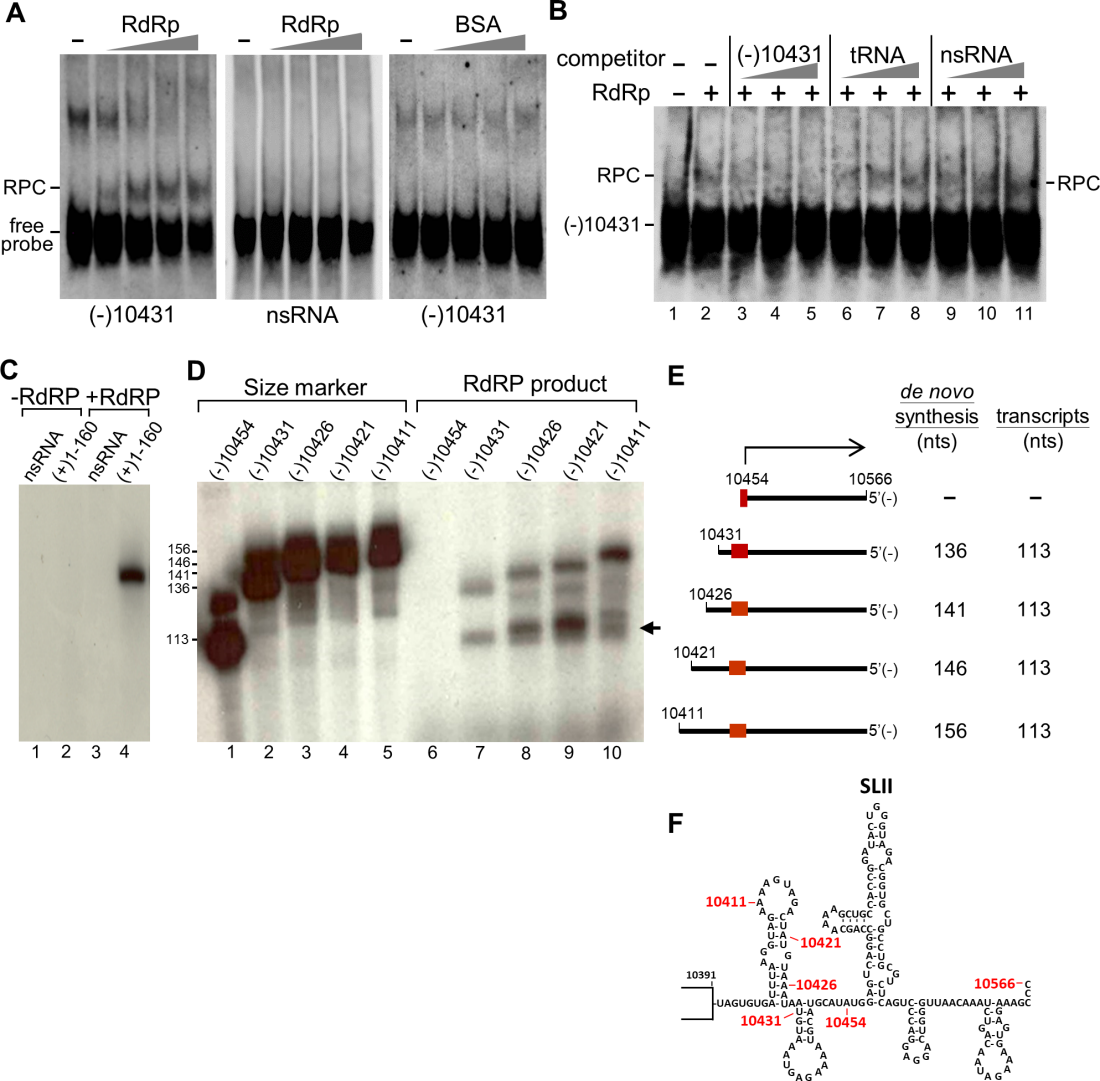
The (-)10431-10566 RNA fragment is a functional template for the initiation of RNA synthesis. (A) EMSA showing the interaction between the (-)10431-10566 RNA with the purified recombinant RdRp protein. Uniformly DIG-labeled (-)10431-10566 RNA (0.25 pmol) or nonspecific RNA (nsRNA) from 5’ 152-nt of bovine coronavirus genome were titrated with increasing concentrations of RdRp (or BSA) from 0.25, 0.5, 1 to 2.5 μM. The complexes were resolved on a 5% native polyacrylamide gel. Position of the free probe and RNA-protein complexes (RPC) are indicated. (B) Specific interaction of RdRp with the (-)10431-10566 RNA. All reactions contained the same amount of DIG-labeled (-)10431-10566 RNA (0.25 pmol). (Lane 1) free probe; (lanes 2–11) 2.5 μM of RdRp were used; (lane 2) no competitor; (lanes 3–11) unlabeled RNA competitors, representing either (-)10431-10566, tRNA, or nsRNA (10-, 50-, 100-fold molar excess) as indicated, were added to the gel-shift assay to compete against complex formation. The complexes were resolved on a 5% native polyacrylamide gel. (C) Template specificity of the purified RdRp. Templates tested were nsRNA and (+)1-160 RNA (the SLA-containing promoter element) as indicated on the top. (D) Transcripts driven by T7 RNA polymerase used as size markers (lanes 1–5) or RNA templates used for *in vitro* RdRp assays (lanes 6–10) are shown on the top. The products were resolved on 6% polyacrylamide gel containing 6M urea. (E) Schematic diagram of the minus RNA fragments used as templates for the *in vitro* RdRp assays. The numbers correspond to the nucleotide positions in the JEV genome. The products are depicted as *de novo* synthesis or transcription products with size (nts) indicated. (F) Diagram of the secondary structure of the 3’-UTR up to nt 10566 with relative position marked for the templates used by *in vitro* RdRp assays.

To explore the possibility that the JEV sfRNA could be a transcription product, we assumed that its promoter would be located on the antigenome adjacent to the 5’ initiation of the sfRNA. To test whether viral RdRp binds to the putative promoter and initiates sfRNA synthesis, we cloned the cDNA fragment corresponding to the nucleotide at position 10431–10566 of the genome, and performed EMSA experiments using the minus-strand RNA as the riboprobe. Purified RdRp protein (0.25, 0.5, 1, and 2.5 μM) retarded the migration of the (-)10431-10566 RNA in a dose-dependent manner, whereas no band shift was observed with non-specific RNA or BSA control (Fig 4A). It should be noted that the additional band above the RNA/protein complex (RPC) (Fig 4A, lanes 1–3) is inversely correlated to the occurring of RPC suggesting that the highly ordered RNA secondary structure may have intermolecular interactions when separated by non-denaturing gel electrophoresis. This band is an indirect evidence of RdRp binding at this region. The intermolecular interactions were disrupted with increasing amounts of RdRp (Fig 4A, left panel). In contrast, the band remained the same when incubated with increasing amounts of BSA control (Fig 4A, right panel). Competition assays were performed to test the specificity of the interaction between the RdRp and the (-)10431-10566 RNA. Increasing amounts of unlabeled RNA out competed the RPC (Fig 4B, lanes 3–5). In contrast, neither tRNA (lanes 6–8) nor nonspecific RNA (lanes 9–11) competed away the RPC. These results suggested that JEV RdRp binds specifically to the (-)10431-10566 RNA.

Studies have shown that the first 160 nucleotides containing the 5’ UTR and 5’CS of the plus-strand RNA in DENV [34] and WNV [35] contain promoter activity. We first tested the enzymatic activity of purified recombinant JEV RdRp using (+)1-160 RNA as a template for an *in vitro* RdRp assay. The result showed that the (+)1-160 RNA is a functional template for RNA synthesis whereas the 5’ 226-nt RNA from bovine coronavirus (as a nonspecific template control) could not direct RNA synthesis, nor the reaction without adding the RdRp (Fig 4C), indicating that the purified recombinant JEV RdRp has specific enzymatic activity. We then used the (-)10431-10566 RNA as the template for the RdRp assay. Two major synthetic products were detected, the larger band with 136 nt in length same as the size marker driven by T7 RNA polymerase, and the smaller band with 113 nts in length presumably a transcription product initiated from the putative promoter (Fig 4D, lane 7). We then constructed various templates for *in vitro* RdRp assays (Fig 4E and 4F and S1 Table). Interestingly, all constructs showed a *de novo* synthesis product same as the template size and a smaller band with approximately 113 nts in length except for the (-)10454-10566 RNA (Fig 4D and 4E). Although some nonspecific bands were observed, these results indicated that the recombinant JEV RdRp could recognize the promoter and synthesize the corresponding sequences.

## Discussion

Several studies have demonstrated that the accumulation of the sfRNA results from incomplete degradation of viral genome by host exoribonuclease XRN1 [24–26]. The crystal structure of the xrRNAs in the 3’ UTR that resist the degradation process has been determined [7, 23], and the function of the stalling has been studied in several flaviviruses [26, 36–38]. Although stalling of XRN1 by the JEV sfRNA has been reported previously [14], detailed mechanism of the sfRNA formation has not yet been elucidated. The results of this study showed that either knocking down or knocking out of XRN1 gave rise to more sfRNA accumulation in virus-infected cells (Fig 1A, 1B and 1D). It has also been reported that XRN1 could be redundant with other exonucleases in producing Zika virus sfRNAs [7]. These controversial results may be explained by several reasons including (i) exonucleases other than XRN1 can contribute to sfRNA formation, (ii) depletion of XRN1 causes changes in total cellular RNA level that may include products of endonucleolytic cleavage, and (iii) reduction in XRN1 may contribute to differences in susceptibility to viral infection [7]. Although we cannot vehemently rule out these possibilities, theoretically, exoribonuclease XRN1 depletion should reduce the production of the sfRNA, resulting in a reduction of the molar ratio of the sfRNA to the genome, yet an increase rather than a decrease in molar ratio was observed. Furthermore, no intermediate degradation byproducts from viral genome to the sfRNA were detected during infection even in the XRN1-deficient cells (Fig 1A–1D). Morevoer, several unsolved mysteries including the unreasonably high rate of nonsense-mediated decay to generate the sfRNA, the question of how the viral genome within the membrane-protected complex is subjected to RNA degradation, and the lack of economy in degradation from the full-length genome from an evolutionary point of view, led us to reconsider other possible mechanisms of the JEV sfRNA formation.

Mutation of the GDD motif of RdRp in JEV halted the accumulation of sfRNA (Fig 3), suggesting that abundant accumulation of sfRNA depends on viral replication. By transfecting replicon constructs containing various deletions Pijlman et al. demonstrated that sfRNA does not require viral RNA replication, viral proteins, or 5’ UTR since all deletion mutants showed similar levels of sfRNA accumulation [8]. The sfRNA was also detected in BHK-21 cells electroporated with nonreplicating replicon DENV-2 containing the catalytic GDD motif of the NS5 replaced with AAA [20]. The initial RNA transcripts either from replicon or infectious clone are all driven by CMV promoter. If formation of the JEV sfRNA were not required for RNA replication, the accumulation of sfRNA could be detected in the GDD-mutant infected cells. Although efficient RNA replication is required for the detection of any flaviviral RNAs no matter the sfRNA is made either by degradation or by transcription, our results are clearly different from those of previous studies of the WNV and DENV. Although the WNV and JEV belong to the same serotype, different viruses may have evolved to have different strategies for survival in infected cells.

**Fig 5:**
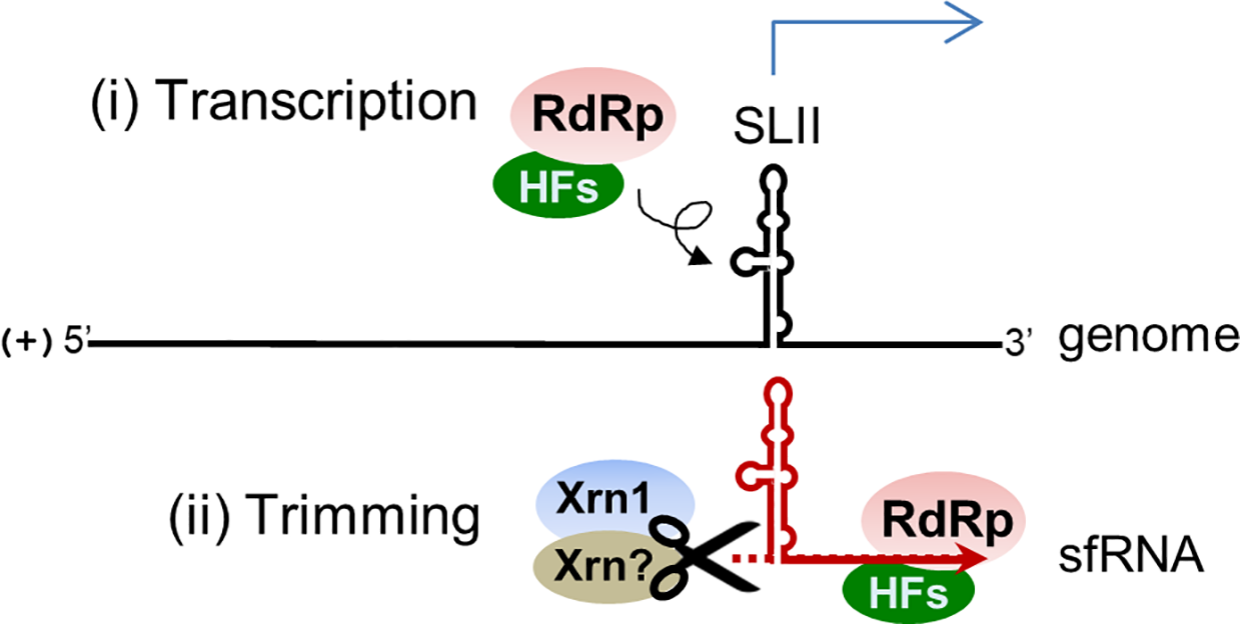
Schematic model depicting the mechanism of JEV sfRNA formation. The JEV sfRNA is likely made (i) by transcription initially by RdRp in conjunction with other host factors (HFs), and (ii) the synthesized products could be further trimmed by exoribonuclease XRN1 and/or other unidentified enzymes.

It is intriguing that, according to *in vitro* RdRp assay results, the NS5 protein binds to the putative promoter and produces a *de novo* synthesis product and a presumable transcription product of approximately 113 nts in length (Fig 4). We attempted to clarify the initiation site of the RdRp products by applying 5’ rapid amplification of cDNA ends (5’ RACE) assays but failed to observe any specific sequences due to low amount of the radiolabeled products. Efforts had also been made to demonstrate that the putative promoter is functional in infected cells by transfecting RNA construct containing unrelated sequences under the control of the promoter. Unfortunately, our numerous attempts to make such a construct failed probably because the transfected molecule may not be accessible as a template to the viral RdRp at the right place and right time. It has been shown that the recombinant JEV NS5 exhibited a weak terminal transferase activity only when substrate ribonucleotides were limited [39]. Sufficient amounts of ribonucleotides were supplied in the *in vitro* RdRp assay (Fig 4C and 4D), thus the products are least likely resulting from the terminal transferase activity. Furthermore, *in vitro* digestion of the 3’ UTR containing fragment (a 800-nt RNA) by XRN1 revealed that XRN1 could stall at a position to generate the sfRNA (Fig 1E). These results let us to propose the model that the JEV sfRNA is likely made by transcription initially and then could be further trimmed by XRN1 or other unidentified exoribonucleases (Fig 5).

Several sequences and structures were determined to define the *cis*-acting elements for sfRNA formation. It has been shown in previous studies with WNV that the deletion of the 5’ half of the 3’ UTR does not affect the genome replication but does halt the sfRNA formation [8]. However, a similar deletion at the 3’ UTR of JEV (Δ307) totally killed virus replication (Fig 2C, lane 3). Whether a partial deletion of the dumbbell structure resulted in the lethal phenotype remains to be determined. It has been suggested that the subgenomic RNA promoter sequences often form a stem-loop structure, which is proposed to facilitate the interaction with the transcriptase as well as other host factors [40]. We found that deletion of the entire or any part of the SLII all impaired sfRNA synthesis (Fig 2C, lanes 7–10), indicating that the SLII structure is essential for sfRNA accumulation and could be a potential promoter for the sfRNA synthesis. Furthermore, pseudoknot structure on SLII is required for the formation of sfRNA in WNV, yellow fever virus (YFV), and ZIKV [7, 24, 25]. Our result also showed that pseudoknot structure is essential for the formation of sfRNA in JEV (Fig 2C, lanes 11–13). Interestingly, mutations made at the SLII region showed relatively smaller plaques and delayed viral maturation or release (Fig 2E and 2F). This observation may correlate with the report that sfRNA-deficient WNV displays significantly decreased infection and transmission rates in mosquitoes [21].

Our results revealed from *in vivo* mutagenesis studies (Fig 2) and *in vitro* RdRp assays (Fig 4) are quite consistent except for the template nt 10454–10566. This template contains the entire SLII region that is essential for the formation of sfRNA *in vivo* (Fig 2) but has no product by the *in vitro* RdRp assay (Fig 4D). This difference may due to that there is not enough space on the template for RdRp binding because RdRp is considered as a large protein so both *de novo* products and transcripts vanished when performed the *in vitro* RdRp assay (Fig 4D, lane 6).

Deletion of the SLII in WNV led to downstream XRN1 stalling and the accumulation of smaller sfRNA species (sfRNA2, sfRNA3 and sfRNA4) [8, 25]. Previous studies of YFV have shown that the sfRNA2 is indeed truncated at the 3’-end [24]. It has been reported that the sfRNA2 only exists in certain isolates of the DENV-2 [6]. Filomatori et al. reported that the accumulation of different species of sfRNAs in DENV-2-infected vertebrate and invertebrate cells due to selective pressures act on the 3’ UTR during host adaption [41]. Although smaller sfRNA species were found in JEV, sequences of these RNAs have not yet been determined. Nevertheless, deletion mutants made on the SLII structure abolished all sfRNA formation but did not give rise to smaller RNA species (Fig 2C). It is still unclear whether the downstream SLIV or the dumbbell structure on JEV genome plays a role in stalling of the XRN1. The mechanism for the formation of smaller JEV RNA species needs to be further characterized.

Genome cyclization has been shown to play an essential role in the replication of flaviviruses (for review see [33]). A promoter element known as stemloop A (SLA) at the 5’ end of the genome has been well studied with regard to its function in DENV and WNV [34, 35, 42]. The initiation of minus-strand RNA synthesis includes the RdRp binding at the SLA at the 5’ end of the genome and the relocation of the RdRp at the 3’ initiation site by long-range RNA-RNA interactions [34, 43, 44]. In this study, we identified the SLII that showed promoter activity as good as the SLA promoter (Figs 2 and 4). We also found that the initiation site of JEV sfRNA located at a 3’-cyclization-like motif. This CYC-like motif complementary to the 5’ cyclization sequences may be involved in genome cyclization during antigenome synthesis and may cause template switching to occur, as reported by Maeda *et al*. [45]. However, deletion of the CYC-like motif had no effect on sfRNA accumulation *in vivo* (Fig 2C, lane 6). A potential role of this CYC-like motif remains to be studied.

Although viruses in the same family share many common strategies for completion of their life cycles, some differences in those strategies make certain viruses unique within the given family. In the present study, we provide evidence that the JEV sfRNA is likely synthesized by transcription and could be further trimmed by exoribonuclease XRN1 and/or other unidentified enzymes (Fig 5). The SLII structure is responsible for sfRNA synthesis. Our observations expand the current knowledge about the mechanism of sfRNA formation in JEV-infected cells.

## Supporting information

S1 Table Synthetic oligonucleotides used in this study. (DOC)

S1 Text Online supplemental materials and methods for constructing plasmids and site-directed mutagenesis. (DOC)
