# Supplementary material for: The conserved stem-loop II structure at the 3' untranslated region of Japanese encephalitis virus genome is required for the formation of subgenomic flaviviral RNA
Source: PLoS One. 2018 Jul 26;13(7):e0201250. doi: 10.1371/journal.pone.0201250 (PMC6062100; doi:10.1371/journal.pone.0201250)
Supplement: S1 Table — (DOC) [file pone.0201250.s001.doc]

**S1 Table.** Synthetic oligonucleotides used in this study.

| Construct | Oligonucleotide^a^ | Sequence (5'-3')^b^ | Binding region^c^ |
| --- | --- | --- | --- |
| RdRp | F1 | cactgtggatccCATAGCAATCAGGAGAAAATCAAG | 8493-8516 |
|  | R1 | tagtcactcgagTCAGATGACCCTGTCTTCCTGG | 10373-10394 |
| *In vitro* RdRp assays | | | |
| (+)1-226 | F2 | *taatacgactcactat*GATTGTGAGCGATTTGCGTGCG | 1-22 (BCoV) |
|  | R2 | gaattcTGTTGATCTTCGACATTGTG | 207-226 (BCoV) |
| | (+)1-160 | F3 | *taatacgactcactatA*GAAGTTTATCTGTGTGAACTT | 1-22 | | --- | --- | --- | --- | | F3 | *taatacgactcactatA*GAAGTTTATCTGTGTGAACTT | 1-22 |
|  | R3 | gaattcGGTAGGCCGCGTTTCAGC | 143-160 |
| (-)10411-  10566 | F4 | aagcttAAAGTAGACTATGTAAATAATGTAAA | 10411-10436 |
|  | R4 | *taatacgactcactata*GGGCTTTCTCACTTTCTATTGTCAGATTTG | 10537-10566 |
| (-)10421-  10566 | F5 | aagcttATGTAAATAATGTAAATGAGAAAATG | 10421-10446 |
| (-)10426-  10566 | F6 | aagcttAATAATGTAAATGAGAAAATGCATGC | 10426-10451 |
| (-)10431-  10566 | F7 | aagcttTGTAAATGAGAAAATGCATGCATATG | 10431-10456 |
| (-)10454-  10566 | F8 | aagcttTGTAAATGAGAAAATGCATGCATATG | 10454-10475 |
| Nuclease assays | | | |
| | pGEMT-JEV-800 | F9 | AGCTGGACAGACGTTCCGTAGT | 10185-10207 | | --- | --- | --- | --- | |  | R9 | AGATCCTGTGTTCTTCCTCA | 10957-10976 | | DENV-800 | F10 | *taatacgactcactatag*TCAGGCTGGCGGCAAATGCT | 9884-9903 | |  | R10 | AGAACCTGTTGATTCAACAGCACCATT | 10697-10723 | | | | |
| Mutagenesis of the infectious clone (pTight-JEV) | | | |
| JEV-9082 | F11 | AAGCTAAAGGAAGCAGGGCC | 9082-9101 |
| Δ307 | F12 | TAGAAAAGTAGACTATCAATAGACGAGGTGTAAGGACT | 10407-10422  10730-10751 |
|  | R12 | TACACCTCGTCTATTGATAGTCTACTTTTCTACCTTAA | 10401-10422  10729-10745 |
| ΔAU-rich | F13 | GAAAAGTAGACTATGTATGAGAAAATGCATGCATATGGA | 10409-10425  10437-10458 |
|  | R13 | GCATGCATTTTCTCATCATAGTCTACTTTTCTACCTTA | 10402-10423  10436-10451 |
| Δ5’-SL | F14 | GAAAAGTAGACTATGTAAATAACATATGGAGTCAGGCC | 10409-10430  10451-10466 |
|  | R14 | GGCCTGACTCCATATGTTATTTACATAGTCTACTTTTC | 10409-10430  10451-10466 |
| ΔCYC-like | F15 | ATGTAAATGAGAAAATGGTCAGGCCAGCAAAAGCTGCCACC | 10430-10446  10459-10482 |
|  | R15 | AGCTTTTGCTGGCCTGACCATTTTCTCATTTACATTATTTAC | 10423-10446  10459-10472 |
| ΔSLII | F16 | AATGCATGCATATGGAGTCTCAGTCCCAGGAGGACTGG | 10443-10458  10510-10531 |
|  | R16 | CTCCTGGGACTGAGACTCCATATGCATGCATTTTCTCA | 10437-10458  10510-10525 |
| ΔSLII.1 | F17 | GCATATGGAGTCAGGCCCACCGGATACTGGGTAGACGGTGC | 10450-10465  10478-10502 |
|  | R17 | ACCCAGTATCCGGTGGGCCTGACTCCATATGCATGCATTTTC | 10440-10465  10478-10493 |
| ΔSLII.2 | F18 | GGCCAGCAAAAGCTGCCTGCCTGCGTCTCAGTCCCAGGA | 10463-10478  10502-10524 |
|  | R18 | ACTGAGACGCAGGCAGGCAGCTTTTGCTGGCCTGACTC | 10457-10478  10502-10517 |
| ΔSLII.3 | F19 | TGCATGCATATGGAGTCAGCAAAAGCTGCCACCGGATA | 10445-10463  10469-10487 |
|  | R19 | GTGGCAGCTTTTGCTGACTCCATATGCATGCATTTTCT | 10439-10460  10466-10481 |
| PK1’ | F20 | AAAGCTGCCACCGGATAgTcGcTAGACGGTGCTGCCTGCGTCTC | 10471-10514 |
|  | R20 | CAGGCAGCACCGTCTAgCgAcTATCCGGTGGCAGCTTTTGCT | 10467-10508 |
| PK1” | F21 | ACGGTGCTGCCTGCGTgTgAcTCCCAGGAGGACTGGGTTAACA | 10496-10538 |
|  | R21 | CCCAGTCCTCCTGGGAgTcAcACGCAGGCAGCACCGTCTACCC | 10490-10532 |
| PK1’1” | F22 | GCTGCCACCGGATAgTcGcTAGACGGTGCTGCCTGCGTgTgAcTCCCAGGAGGACTGGG | 10474-10532 |
|  | R22 | GTCCTCCTGGGAgTcAcACGCAGGCAGCACCGTCTAgCgAcTATCCGGTGGCAGCTTTTG | 10469-10528 |
| HDV-  Ribozyme-R | R23 | cttagccatccgagtggacgtgc | 823-845 (HDV) |

^a^Oligonucleotide with plus or minus sense is indicated by (F) or (R), respectively.

^b^Upper case letters denote JEV sequences and lowercase letters denote non-JEV sequences. The underlined sequences represent restriction endonuclease sites and italics represent the T7 promoter.

^c^Most numbers correspond to nucleotide positions in the positive strand JEV PR9 genome (in GenBank under accession number AF014161). The F2 and R2 primers bind to bovine coronavirus genome (BCoV, accession number U00735), the F10 and R10 primers bind to DENV-2 16681 genome (accession number KU725663), and the R23 primer location corresponds to hepatitis D virus (HDV, accession number NC001653) as indicated.
