# Supplementary material for: The conserved stem-loop II structure at the 3' untranslated region of Japanese encephalitis virus genome is required for the formation of subgenomic flaviviral RNA
Source: PLoS One. 2018 Jul 26;13(7):e0201250. doi: 10.1371/journal.pone.0201250 (PMC6062100; doi:10.1371/journal.pone.0201250)
Supplement: S1 Text — (DOC) [file pone.0201250.s002.doc]

**S1 Text**

**Plasmids and site-directed mutagenesis**

Plasmids used for this study were constructed mainly by RT-PCR using synthetic oligonucleotides as listed in S1 Table. T7 promoter sequences as well as unique restriction sites were incorporated in either forward (F) or reverse (R) primers designed to amplify specific regions for run-off transcription. Non-specific control RNA used for EMSA analysis was *in vitro* transcribed from *HpaI*-linearized pDrep1 (JVI 68:8223, 1994). Infectious cDNA clone was conducted as described previously (JGV 95:1493, 2014).The replication-deficient mutant, which had the essential polymerase motif GDD mutated to a G (NS5mt), was constructed by homologous recombination of three fragments in yeast cells (YPH857): (i) the *Aat*II-linearized pTight-JEV fragment containing the 5’-UTR-C-prM, the 3’-UTR, and the vector sequences; (ii) the *Pme*I and *Xma*I linearized pTight-JEV fragment containing the C through the NS2A sequences; and (iii) the *Xho* I and *Hpa* I digested replicon (SP6-JC107-RL-GDD-M) containing the E throughout the 3’UTR with the mutation in NS5. Recombinant mutants were puriﬁed from yeast cells and re-ampliﬁed in *E. coli* strain C41. Various mutations were introduced into the WT full-length cDNA (pTight-JEV) by replacing the *Xba*I-*Rsr*II fragment of the pTight-JEV DNA plasmid with a fragment derived from an overlap PCR mutagenesis procedure containing the desired mutations. For each of these mutants, two gel purified PCR products, one from a pTight-JEV-templated reaction with primers F11 and corresponding reverse primer (R) and the other from a pTight-JEV-templated reaction with primers corresponding forward (F) and R23 were used together. The two overlapping fragments were then amplified with primers F11 and R23 to form a 1974-nt PCR product from which the mutated fragment was used to replace the corresponding region in pTight-JEV by homologous recombination in yeast. Recombinant mutants were puriﬁed from yeast cells, re-ampliﬁed in *E. coli* strain C41. All clones were validated by DNA sequencing. Detailed cloning procedures are available upon request.
